# *SOD2* contributes to anti-oxidative capacity in rabbit corneal endothelial cells

**Published:** 2011-09-24

**Authors:** Cailing Liu, Diego Ogando, Joseph A. Bonanno

**Affiliations:** School of Optometry, Indiana University, Bloomington, IN

## Abstract

**Purpose:**

Corneal endothelial cells are rich in mitochondria, a potential source of reactive oxygen species (ROS). ROS have been implicated in endothelial cell loss during aging or in endothelial dystrophies. In this study we examined the anti-oxidative role of mitochondrial superoxide dismutase (SOD2) in corneal endothelial cells.

**Methods:**

SOD2 expression was examined by RT–PCR and western blot analysis in fresh rabbit corneal endothelium (RCE) and cell cultures. SOD2 activity, total reactive oxygen species (ROS), mitochondrial ROS, mitochondrial membrane potential (MMP), and apoptotic levels were examined in untreated, *SOD2* siRNA and viral vector shRNA treated RCE cells. Scrambled siRNA and shRNA sequence targeting non-mammalian genes were used as controls.

**Results:**

*SOD2* is expressed in both fresh and cultured rabbit corneal endothelium. *SOD2* expression was reduced by ~80%–90% in cultured RCE using either siRNA or shRNA approaches. *SOD2* activity was decreased by ~70%–80% for both approaches. Total cell ROS was significantly increased in shSOD2 lentivirus treated cells (9%±6%) relative to control transduction (0.4%±0.1%). MitoSOX™ staining for mitochondrial ROS in siSOD2 treated RCE cells was dramatically increased. Two minutes of UV irradiation increased total ROS levels by 15%, whereas in shSOD2 treated cells UV induced ROS was increased 29%±5% (p<0.05). MMP was reduced in shSOD2 viral treated cells by 66%±3%, significantly greater than in control transduced cells (15%±8%, p<0.05). Apoptosis increased by 1.5 fold in shSOD2 virus treated samples compared with scrambled virus and untreated cells.

**Conclusions:**

SOD2 is expressed in both fresh and cultured rabbit corneal endothelium. siRNA and shRNA approaches are able to efficiently knockdown SOD2 expression and reduce enzyme activity in RCE cells. Decreased SOD2 activity causes elevated ROS production, mitochondrial membrane potential loss and early cell apoptosis. These results indicate that SOD2 is a significant anti-oxidative enzyme in RCE cells.

## Introduction

Corneal endothelium (CE) is a monolayer of cells on the posterior surface of the cornea. The primary function of the corneal endothelium is to maintain corneal transparency by controlling stromal hydration. In humans, CE cells have limited repair capacity. Therefore, cell loss or damage caused by aging, disease or injury is permanent. Oxidative stress is present in the cornea because exposure to light is a significant source for reactive oxygen species (ROS) [1-3]. Moreover, CE cells are rich in mitochondria, which is a potential source for superoxide production. Under normal physiologic conditions, nearly 4% [4] of the oxygen consumed by the electron transport chain in mitochondria is converted to 2–3 nmol per min per milligram protein superoxide anions [5] as an inevitable byproduct of oxidative metabolism. Oxidation of mitochondrial lipids, proteins, and DNA (mtDNA) can cause cell damage. These features make the corneal endothelium particularly susceptible to oxidative stress.

ROS toxicity is reduced predominantly through antioxidant enzymes including superoxide dismutase (SOD), catalase, glutathione peroxidase (GPx) and glutathione transferase. Expression of anti-oxidative genes is prominent in ocular tissues [6-12]. As primary antioxidant systems, SOD isoenzymes, catalyze the dismutation of the superoxide radicals to generate hydrogen peroxide. Copper/Zinc SOD encoded by SOD1 exists in the cytosol. Manganese SOD encoded by SOD2 is in the mitochondrial matrix and secretory SOD, encoded by SOD3 is distributed in the extracellular space. This compartmentalization is needed because the superoxide anion radical exerts its effects locally and the anion poorly penetrates membranes [11].

Corneal endothelial cells are second only to photoreceptors for the highest aerobic metabolic rate of all cells in the eye [13]. Consistent with the high oxygen consumption, there is growing evidence to support the hypothesis that ROS toxicity contributes to endothelial loss in aging and disease. For example, in Fuchs corneal endothelial dystrophy, a condition that shows accelerated cell loss, SOD2 expression is significantly suppressed and there is evidence of significant oxidative damage [14]. Surprisingly, no compensatory increase in the level of other antioxidative enzymes, such as catalase or glutathione peroxidases (GPx) and/or transferase, is observed [14].

*SOD2* knockouts are lethal [15]. Therefore, molecular approaches to changing SOD2 activity have relied on partial knockouts [16-18], RNAi knockdown [19-21], or overexpression [18,22-25]. For example, SOD2 protects mouse retinal pigment epithelium (RPE) from oxidative challenge [18]. Knockdown of *SOD2* activity in mouse RPE induced early stages of age related macular degeneration (AMD) changes and can be used as an animal model for studying AMD [20]. Given these features of corneal endothelial cells and the important anti-oxidative role of SOD2, we hypothesize that SOD2 plays a significant anti-oxidant role in CE cells.

## Methods

### Cell culture

Rabbit corneal endothelial (RCE) cells were isolated from fresh peeled corneal endothelium of young (8 weeks) New Zealand white rabbit eyes (Pel-Frez Biologicals, Roger, AR). Peeled endothelium was incubated in disaggregating solution (300 Units type I collagenase, 100 Units hyalronidase, and 1% antibiotic/antimycotic) in Dulbecco’s Modified Eagle Medium (DMEM; Invitrogen, Carlsbad, CA) for about 3 h at 37 °C. CE cells were collected by centrifugation at 450× g for 5 min. Cells from four corneas were suspended in a 35 mm Petri dish with 2 ml DMEM supplemented with 10% bovine calf serum (Thermo Scientific, Logan, UT) and 1% antibiotic-antimycotic. Cells were incubated at 37 °C with 5% CO_2_ and passaged 3 days later when they reached 90% confluence.

### siSOD2 design and transfection

Two sense and antisense oligonucleotides corresponding to *Oryctolagus cuniculus* (rabbit) manganese superoxide dismutase (*SOD2*) mRNA (Genebank L28808.1, 5′-AAA CGT CAG ACC TGA TTA TCT-3′ (525–545) and 5′-AAT GTA ACT GAA AGA TAC ATG-3′ (577–597) were designed by an Ambion siRNA targeting design tool (Ambion, Austin, TX) and blasted by NCBI_BLAST. siSOD2 was synthesized using the Silencer siRNA construction kit (Ambion, Austin, TX). An siControl nontargeting siRNA (no known mammalian homology) was purchased from Invitrogen (Carlsbad, CA). Fifty thousand cells/well were seeded in 12-well plates and transfected with 10 nM siSOD2 or siControl by using Lipofectamine^TM^ 2000 (Invitrogen). Transfected cells were incubated with 1 ml of Opti-MEM (Invitrogen) containing siRNA for 4 h, followed by addition of 1 ml of standard DMEM with 2% serum. Transfected cells were harvested 68 h post transfection for detecting knockdown efficiency by western blot, enzyme activity assay, and ROS production assay. Based on *SOD2* knockdown results, a sequence targeting 5′-AAA CGT CAG ACC TGA TTA TCT-3′ (525–545) was selected for the remainder of the experiments and used for shSOD2 design.

### shSOD2 pseudo-lentivirus production and transduction

Lentiviral vectors were produced to test the relative knockdown efficacy with an eye to using them in future in vivo applications. shSOD2 was designed following the manufacturer’s (System Biosciences, SBI, Mountain View, CA) instructions with primers: 5′-GAT CCA AAC GTC AGA CCT GAT TAT CTC TTC CTG TCA GAA GAT AAT CAG GTC TGA CGT TTT TTT TG-3′ and 5'-AAT TCA AAA AAA ACG TCA GAC CTG ATT ATC TTC TGA CAG GAA GAG ATA ATC AGG TCT GAC GTT TG-3'. An shRNA targeting wild type firefly Luciferase gene was designed as a scrambled control (5′-GAT CCG TGC GTT GTT AGT ACT AAT CCT ATT TGT GAA GCA GAT GAA ATA GGG TTG GTA CTA GCA ACG CAC TTT TTG-3′ and 5′-AAT TCA AAA AGT GCG TTG CTA GTA CCA ACC CTA TTT CAT CTG CTT CAC AAA TAG GAT TAG TAC TAA CAA CGC ACG-3′). shSOD2 and shLuciferase template oligonucleotides were synthesized (Integrated DNA Technologies, IDT, Coralville, IA) and cloned into feline immunodeficiency virus (FIV) based pSIF-H1-Puro shRNA vectors (SBI). As described previously [26], lentiviral constructs pSIF-H1-Puro-shSOD2 and pSIF-H1-Puro-shLuciferase were individually packaged into lentiviral transducing particles which were pseudotyped with vesicular stomatitis virus G (VSV-G) protein (pPACK ^TM^ lentivector packaging kit, SBI). Briefly, lentivirus vector was combined with the mixture of pFIV-34N and pVSV-G plasmids, and then transfected into 293TN HEK cell by using Lipofectamine ^TM^ Reagent (Invitrogen). Pseudovirus containing medium was collected 48 and 72 h post transfection followed by purifying pseudo-lentiviral particles with PEG*-it* virus precipitation solution (SBI) overnight at 4 °C. The viral particle preparation was centrifuged at 1,500× g for 30 min at 4 °C. Residual PEG*-it* solution was removed by another centrifugation at 1,500× g for 5 min. Viral particles were washed and then resuspended in cold, sterile phosphate buffered saline (PBS). Virus stocks were aliquoted and stored at −80 °C. To determine virus titer, serially dilutions of stocks were inoculated to 293TN HEK cells. Seventy-two hours post viral transduction, cells were collected for a real time PCR-based titration (UltraRapid Lentiviral Titer Kit; SBI) based on the amplification of a small fragment from the lentivector-specific WPRE (Woodchuck hepatits virus post transcriptional Regulation Element) that is integrated into the genome of transduced cells.

Fifty thousand RCE cells/well were seed in 12-well plates overnight and transducted with shRNA virus at an m.o.i. of 20 in the presence of 7 mg/ml of Polybrene (Hexadimethrine Bromide; Sigma, St. Louis, MO). Cells were collected at 5 days post transduction for western blot analysis, enzyme activity assay, ROS production assay, mitochondrial membrane potential and cell apoptosis assay.

### RT–PCR

Total RNA was extracted from freshly dissected corneal endothelium or cultured RCE using an RNeasy Micro Kit (Qiagen, Valencia, CA). RT–PCR was performed using SuperScript™ One-Step RT–PCR with Platinum® *Taq* (Invitrogen) with the primers of 5′-CTC CCC GAC CTG CCC TAC GAC-3' and 5'-TGC AGG TAG TAA GCG TG-3'. RT–PCR reactions comprised 30 min reverse transcription at 50 °C for 30 min, 2 min of initial denaturation at 94 °C. 40 cycles of denature (94 °C for 15 s), annealing (58 °C for 30 s) and extension (72 ^o^ C for 1 min), followed by a 5-min final extension at 72 °C.

### Western blot

SOD2 protein expression level was determined by western blot analysis as previously described [26], β-actin in the same sample was detected as an internal control. RCE cells were washed with cold PBS and then extracted with RIPA lysis buffer (150 mM NaCl, 0.1% SDS, 0.5% sodium deoxycholate, 1% NP-40) with complete protease inhibitor cocktails (Sigma, St Louis, MO). The preparation was briefly sonicated (Branson Sonifier 250, Dunbury, CT) on ice and centrifuged at 10,000× g for 5 min. An aliquot of the supernatant was taken for protein concentration measurement using the BCA protein assay (Bio-Rad, Hercules, CA). Samples were separated on SDS–PAGE gels and electroblotted to polyvinylidene difluoride (PVDF) membranes (Bio-Rad). Based on the prestained protein markers (Precision Plus protein standards; Bio-RAD), blots were horizontally cut into two parts just above the 37 kDa prestained marker. The lower part with protein sizes less than 37 kDa was probed with SOD2 polyclonal antibodies (Millipore/Upstate, Billerica, MA) at 1:2,000. The upper part was probed with β-actin monoclonal antibody (Sigma) at a dilution of 1:10,000. Enhanced chemiluminescence (ECL, Thermo Scientific/Pierce, Rockford, IL) was used for detection. Films were scanned to produce digital images that were then subjected to densitometry analysis (UN-SCAN-IT gel 6.1; Silk Scientific Corporation, Orem, UT). SOD2 protein levels in test samples were normalized with β-actin (ratio of SOD2 to β-actin). The mitochondrial/cytosolic fractions from RCE cells were separated using a fractionation kit (BioVision, Mountain View, CA). The same amount of protein from each fraction was loaded for western blot analysis. The molecular weight for SOD2 and β-actin is 24 kDa and 43 kDa, respectively.

### Enzyme activity assay

RCE cells were collected in ice-cold PBS (pH 7.2) that contained a protease inhibitor cocktail (Sigma) by centrifugation for 10 min at 250× g at 4 °C. Cells were briefly sonicated on ice and centrifuged for 5 min at 12,000× g at 4 °C. The supernatant was used immediately for activity assay. Protein concentration was measured by BCA protein assay kit. SOD2 activity was detected by using a superoxide dismutase assay kit (Cell Technology, Mountain view, CA). This assay utilizes a highly water-soluble tetrazolium salt, WST-1 (2-(4-Iodophenyl) - 3-(4-nitrophenyl)-5-(2,4-disulfophenyl)- 2H-tetrazolium, monosodium salt) that produces a water-soluble formazan dye upon reduction with a superoxide anion. The rate of the reduction with superoxide anion is linearly related to the xanthine oxidase (XO) activity, and is inhibited by SOD. Reduction in the appearance of WST-1-formazan is a measure of SOD activity present in the experimental samples. WST-1 reduction in siRNA- or shRNA- treated cells was compared to that in siControl- or scrambled shRNA-treated cells, this ratio was calculated as the relative SOD2 activity. Each reaction contained 500 ng protein sample, 600 μl WST-1, 30 μl XO, 1 mM KCN, add XO buffer to a final volume of 800 μl. The reaction without cell lysate was used as a blank control. Each reaction was performed in 40 min at 37 °C. The initial rate of reduction in the appearance of WST-1-formazan was followed by measuring the A_440_ using ND-1000 spectrophotometer (NanoDrop, Wilmington, DE). One millimolar KCN was used to block the activity of SOD1 and SOD3.

### ROS production assay

Mitochondrial ROS in siSOD2 and siControl transfected cells was compared by monitoring MitoSOX™ (Invitrogen/Molecular Probes, Eugene, OR) fluorescence intensity. Ten thousand cells were seeded on a coverslip in a six-well plate and grown overnight. Cells were loaded with 1 µM MitoSOX™ and incubated at 37 °C for 10 min followed by three washes with warm PBS. MitoSOX™ intensity was recorded with a standard epifluorescence microscope (E600; Nikon, Tokyo, Japan) equipped with a cooled charge-coupled device camera (Princeton Instruments, Princeton, NY).

ROS production in shRNA lentiviral treated cells was measured by flow cytometry. Five days post transduction, cells were washed with Hanks’ Balanced Salt Solution (HBSS, Invitrogen) and then loaded with 5 µM 5-(and-6) - carboxy-2′, 7′-dichlorodihydrofluorescein diacetate (carboxy-H_2_DCFDA; Invitrogen/Molecular Probes) and incubated at 37 °C for 30 min. Cells were detached by 0.25% trypsin and then spun at 500× g for 5 min followed by suspending in FACS buffer for measurement. The percentage of carboxy-H_2_DCF stained cells was analyzed by a FACS-Calibur flow cytometer (BD biosciences, San Jose, CA) using CellQuestPro (BD Biosciences) software. A positive control for ROS production was produced by exposing CE to a UV germicidal lamp for 2 min in a laminar biosafety carbinet (Labconco, Janesville, WI). A representative line histogram is graphed by flowJo (version7.6; BD Biosciences) to show the DCF fluorescence intensity shift.

### Mitochondrial membrane potential (MMP) and cell apoptosis assay

MMP was determined with 5, 5′, 6, 6′-tetrachloro-1, 1′, 3, 3′-tetraethylbenzimidazole carbocyanide iodine (JC-1; Invitrogen/Molecular Probes) by using a BMG FLUOstar microplate reader (BMG, Alexandria, VA). Fifty thousand cells per well were grown overnight in 12-well plates in DMEM media. Cells were loaded with 100 nM JC-1 and incubated at 37 °C for 15 min followed by wash with warm HBSS solution. JC-1 was excited at 488 nm, and emission was determined at 529 and 585 nm.

Cell apoptosis was measured by using Annexin V-FITC (BioVision). Annexin V-FITC (5 μl) was added to each well and incubated at room temperature for 5 min in the dark. Fluorescence was measured by microplate reader with ex/em of 488/530. Cells were then collected for BCA protein assay (Thermo scientific/Pierce) to normalize fluorescence per microgram cell protein.

### Statistical analysis

Results are expressed as means±SEM from at least three replicates. Data was analyzed by a two-tailed Student’s *t*-test and one-way ANOVA analysis. A p value <0.05 was regarded as a significant difference.

## Results

### SOD2 expression and knockdown

**Figure 1:**
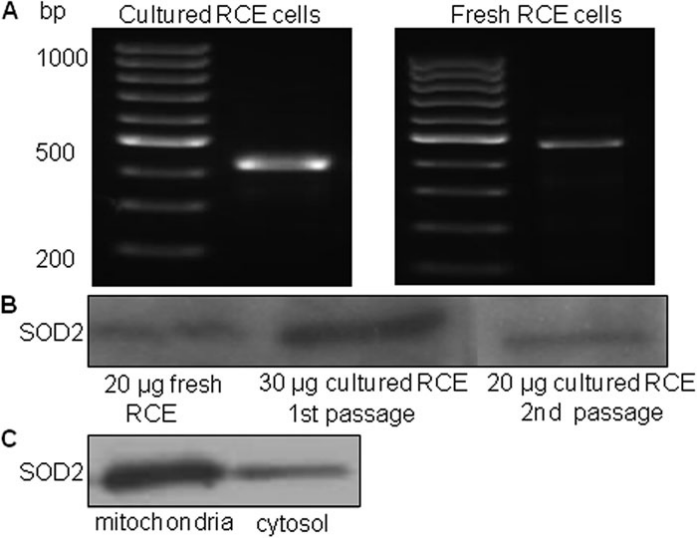
*SOD2* expression in RCE cells. *SOD2* expression was examined by RT–PCR (**A**) and western blot analysis (**B**). SOD2 abundance was compared in mitochondrial and cytosolic fractions by western blot (**C**). A dilution of 1:1,000 of anti-SOD2 was used.

RT–PCR (Figure 1A) and western blot analysis (Figure 1B) confirmed that *SOD*2 is expressed in both fresh and cultured rabbit corneal endothelial cells. Moreover, *SOD2* expression is maintained from passage one to passage two in cultured cells. Figure 1C shows that SOD2 is predominately associated with the mitochondrial fraction.

**Figure 2:**
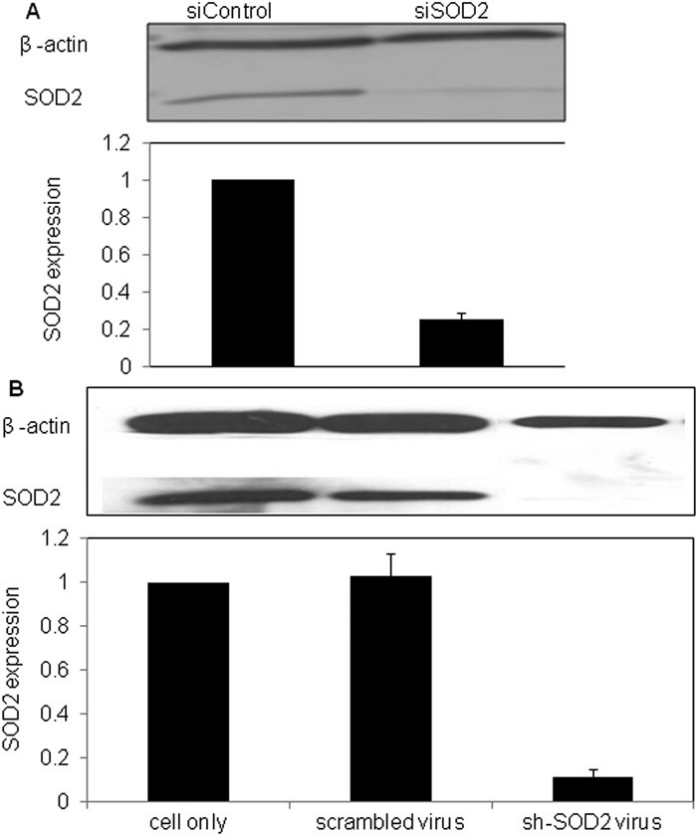
Knockdown of *SOD2* expression in RCE cells. Western blot analysis of *SOD2* knockdown efficiency in siRNA treated (**A**) and shRNA virus treated (**B**) RCE cells. Representative western blot images are shown for each approach.

Two siSOD2 sequences were tested to knockdown rabbit *SOD2* mRNA (Genebank ID L28808.1): 5′-AAA CGT CAG ACC TGA TTA TCT-3′ (525–545) and 5′-AAT GTA ACT GAA AGA TAC ATG-3′ (577–597). Western blot analysis showed that the sequence 5′-AAA CGT CAG ACC TGA TTA TCT-3′ could efficiently knockdown *SOD2* expression with a reduction of 80% in corneal endothelial cells (Figure 2A). This sequence was used for the remaining experiments and shSOD2 design. Primary cultured RCE cells were transfected with shSOD2 lentiviral pseduoparticles with an m.o.i of 20. Five days after viral transduction, cells were collected for detecting *SOD2* expression level. Western blot analysis demonstrated that shSOD2 lentiviral transduction is able to reduce *SOD2* expression by ~90% in cultured cells (Figure 2B).

### SOD2 enzyme activity

**Figure 3:**
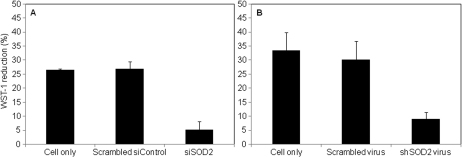
SOD2 enzyme activity assay. SOD2 enzyme activity was determined by using a colorimetric based superoxide dismutase assay kit (Cell Technology). **A** and **B** show the summarized SOD2 activity for siRNA treatment and shRNA treatment, respectively. Preparations from scrambled RNA treated RCE cells reduced WST-1 formazan absorbance by 27%±4% (siControl) and 30%±6% (scrambled virus) relative to the blank, whereas preparations from siSOD2 and shSOD2 lentiviral treated RCE cells reduced absorbance by only 5%±3% and 9%±2%, respectively. WST-1 reduction in siRNA- or shRNA- treated cells was compared to that in siControl- or scrambled shRNA-treated cells, this ratio was calculated as the relative SOD2 activity.

To test the effect of *SOD2* knockdown on enzyme activity, SOD2 enzyme activity was measured by monitoring the change of substrate WST-1 formazan absorbance. Figure 3 shows that SOD2 activity was not significantly different between untreated cells and scrambled RNA treated RCE. However, SOD2 activity was reduced by 81% and 71% in siSOD2 and shSOD2-lentiviral treated cells, respectively.

### ROS production

**Figure 4:**
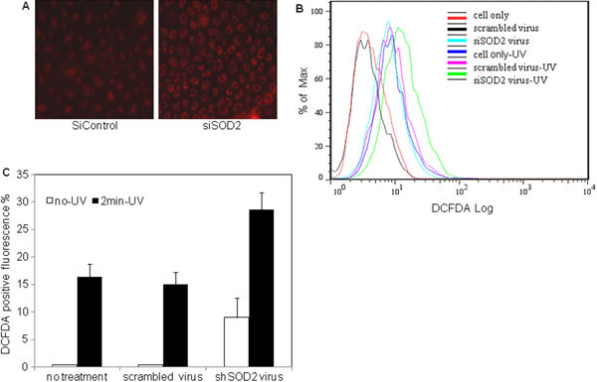
ROS production assay. **A**: MitoSOX™ microscopy ROS detection for siRNA treated RCE cells (200×). **B**: A representative histogram of ROS production for control, scrambled virus-treated and shRNA-treated cells with and without 2 min UV exposure. An increase in relative fluorescence is reflected with a rightwards shift in x-axis in line histograms. The % of Max is the number of cells in each bin divided by the number of cells in the bin that contains the largest number of cells. **C**: Summary data for DCF flow cytometry ROS detection.

Next we asked whether a reduced *SOD2* activity affects ROS levels. Mitochondrial ROS was qualitatively detected by MitoSOX™ staining and total ROS by DCF flow cytometry approaches. Figure 4A shows that MitotSox™ fluorescence intensity in siSOD2 treated RCE cells was dramatically increased relative to that in scrambled sequence treated cells. Figure 4B is a representative histogram and shows that total ROS, as measured by DCF fluorescence also increased following *SOD2* knockdown. The DCF fluorescence intensity shift to the right in shSOD2 virus treated cells indicates an increased ROS production. Figure 4C summarizes DCF flow cytometry analysis. Significant DCF fluorescence was present in 0.4%±0.1% of untreated cells and scrambled-sequence virus treated cells. However, DCF fluorescence positive cells significantly increased to 9%±6% in shSOD2 virus treated cells (p<0.05), a 22.5 fold increase.

Next, we examined the role of *SOD2* in clearing ROS following a burst of ROS production induced by 2 min exposure to a UV germicidal lamp. With 2 min UV irradiation, untreated cells and scrambled sequence treated cells yielded similar levels of DCF positive fluorescence (~15%, Figure 4C), whereas shSOD2 treated cells showed dramatically increased DCF fluorescence (29%±5%, p<0.05). Flow cytometry results indicated that *SOD2* knockdown contributed to ROS generation in RCE cells, while increasing sensitivity to oxidative stress (UV).

### Mitochondrial membrane potential (MMP) and cell apoptosis

**Figure 5:**
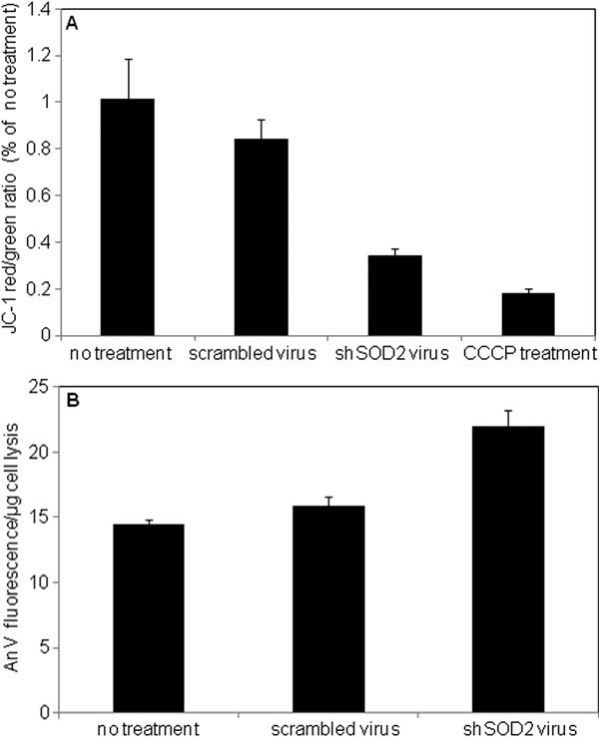
Mitochondrial membrane potential (MMP) and cell apoptosis assay. **A**: Summarized data for MMP change for shRNA treated RCE cells. The ratio of JC-1 red/green fluorescence represents the MMP. y-axis is the ratio of JC-1 red to green, which reflects MMP. Data are normalized with no treatment. MMP for shRNA treated cells relative to that of no treatment (NT). **B**: Summarized data from the Annexin V-FITC (AnV) cell apoptosis assay results. Annexin V-FITC intensity was normalized to protein concentration.

The effect of *SOD2* knockdown on MMP was detected with the cationic dye JC-1. In the undamaged mitochondria with normal membrane potential, JC-1 aggregates produce a red fluorescence, whereas in cells with depolarized MMP, the dye remains as monomers producing a diffuse green fluorescence. The ratio of red/green fluorescence is a measure of MMP, with a decrease in the ratio indicating depolarization. Figure 5A shows that five days post viral transduction MMP in scrambled virus treated cells was about 15%±8% lower than untreated cells. However in shSOD2 viral treated cells MMP was depolarized by 66%±3% (p<0.05). As a positive control MMP was depolarized by treatment with CCCP, a protonophore. This produced 88%±2% decrease in JC-1 fluorescence ratio. These results indicate that the decreased level of *SOD2* expression indirectly induces a depolarization of MMP.

Cellular redox state plays a critical role in programmed cell death. An increased ROS level frequently represents a triggering event upstream of the mitochondrial membrane depolarization, cytochrome c release, caspase activation, and nuclear fragmentation [5,27]. Our results showed that knockdown of *SOD2* elevated ROS production and induced mitochondrial membrane potential depolarization suggesting that *SOD2* knockdown would increase cell apoptosis. shRNA virus treated cells were collected at 5 days post virus transduction for detecting early apoptosis using Annexin V-FITC staining. Figure 5B shows that this assay detected a significantly higher level (1.5 fold, p<0.05) of Annexin V-FITC fluorescence in shSOD2 virus treated samples compared with scrambled virus and untreated cells, indicating that a reduced SOD2 level contributes to early apoptosis in RCE cells.

## Discussion

In the present study, we report for the first time that *SOD2* contributes to anti-oxidative capacity in rabbit corneal endothelial cells. Our results showed that *SOD2* is abundantly expressed in rabbit corneal endothelial cells at mRNA (Figure 1A) and protein (Figure 1B) levels. As expected, SOD2 is predominantly associated with the mitochondrial fraction (Figure 1C). We used chemically synthesized siSOD2 and lentiviral mediated shSOD2 approaches to suppress *SOD2* with about 80% reduction in protein expression (Figure 2) and enzyme activity (Figure 3) in either approach. These results allowed us to further determine if decreased *SOD2* expression and activity contribute to oxidative stress and its consequence. Detection of ROS production showed mitochondrial and total ROS were dramatically increased in CE cells with reduced *SOD2* expression, indicating that *SOD2* is an important anti-oxidative enzyme in CE. Further tests on MMP and cell apoptosis showed that decreased *SOD2* expression and activity result in increased MMP depolarization and cell apoptosis. Taking all these findings together, our results indicate that *SOD2* plays a significant role in protecting CE cells from mitochondrial oxidative stress.

To determine the role of SOD2 as an anti-oxidative protective enzyme in CE cells, we need to alter *SOD2* expression and/or enzyme activity. Since *SOD2* knockout mice showed lethality, overexpression or knockdown of *SOD2* are the candidate choices for this purpose. Overexpression and RNAi approaches manipulate target gene expression exogenously and endogenously, respectively. Overexpression of *SOD2* has been widely used in retinal pigment epithelial cells studies [18,24,25]. However, there are no reports on manipulating *SOD2* expression in corneal endothelial cells. In a previous study, chemically synthesized siRNA has been successfully used in SOD2 functional studies in retina ganglion cells with over 90% knockdown efficiency [21]. In our study we used both siRNA and shRNA approaches. siRNA suppresses gene expression by transient gene knockdown and gives a short knockdown time window, whereas the lentiviral delivered shRNA produces sustained knockdown [26]. Our results show that both approaches can knockdown ~80%–90% of *SOD2* expression, which is similar to that found for retinal ganglion cells by using siRNA [21]. Given the utility of the lentiviral shRNA approach, future in vivo studies can examine the long-term role of endogenous SOD2 in maintaining CE integrity [26].

SOD2 is a crucial scavenger for superoxide in mitochondria [27,28]. Its significance is best demonstrated in *SOD2* knockouts (*Sod2*^tm1Cje^ −/−), which show neonatal lethality [15] that is not reversed or delayed by *SOD1* overexpression [29]. These animals also have a dramatic increase in DNA oxidation products and marked reductions in mitochondrial enzyme activities [30]. Initial studies of partially *SOD2* deficient mice, *Sod2*^tm1Cje^ heterozygote (+/−), revealed increased sensitivity to apoptosis [16], whereas *SOD2* overexpression has an antiapoptotic effect [18,22,23]. SOD2 in corneal endothelial cells appears to have a similar role. Reducing endogenous activity increased the basal level of apoptotic cells and made CE cells more susceptible to exogenous UV stress.

SOD2 converts superoxide to H_2_O_2_ and mitochondrial glutathione peroxidase GPx1 catalyzes the reduction of H_2_O_2_ to H_2_O. Hydroxyl radical is another extremely harmful reactive free radical in mitochondria, formed by combination of superoxide and H_2_O_2._ GPx1 could be another crucial antioxidant enzyme in CE cells. The important synergy between SOD2 and GPx1 has been demonstrated in double heterozygote knockouts [17]. In the future, it would be very interesting to study the synergic antioxidant effort of SOD2 and GPx1 in CE cells.

In summary, by knocking down *SOD2* expression, we provide evidence that *SOD2* is essential for neutralizing endogenous ROS in CE cells. SOD2 activity reduces ROS levels in RCE cells, helps maintain MMP, buffers against acute ROS generation, and reduces cell apoptosis. We conclude that *SOD2* plays a key role as an anti-oxidant in CE cells and maintaining mitochondrial integrity.
